# The clinical evaluation of electroacupuncture combined with mindfulness meditation for overweight and obesity: study protocol for a randomized sham-controlled clinical trial

**DOI:** 10.1186/s13063-022-06725-8

**Published:** 2022-09-27

**Authors:** Ching Yee Chung, Angela Wei Hong Yang, Alexander Foe, Mingdi Li, George Binh Lenon

**Affiliations:** 1 Discipline of Chinese Medicine, School of Health and Biomedical Sciences, RMIT University, PO Box 71, Bundoora, VIC 3083 Australia; 2 Department of Preventative and Health Care, Qingdao Traditional Chinese Medicine Hospital (Qingdao Hiser Hospital), Qingdao, Shandong China

**Keywords:** Obesity, Overweight, Acupuncture, Electroacupuncture, Mindfulness meditation, Mindfulness intervention, Evidence-based Chinese medicine

## Abstract

**Background:**

Overweight and obesity have reached an epidemic level which impacts individual health and creates a financial burden worldwide. Evidence has shown that electroacupuncture is effective for weight loss when combined with lifestyle intervention, while mindfulness meditation can enhance the outcome of weight loss programs. This study aims to evaluate the safety and the add-on effect of electroacupuncture and mindfulness meditation for weight management in overweight and obesity.

**Methods/design:**

This is a sham-controlled, three-armed randomized clinical trial. A total of 165 participants with BMI between 25 and 39.99 and aged between 18 and 60 who meet the inclusion and exclusion criteria will be randomized into [1] electroacupuncture plus mindfulness meditation group, [2] sham electroacupuncture plus mindfulness meditation group, and [3] electroacupuncture only group. The total duration of this study will be 22 weeks, which consists of a 2-week run-in period, a 12-week intervention period, and an 8-week follow-up period. Participants will receive 12 weekly treatments during the intervention period. Primary outcomes will include body mass index, waist and hip ratio, and body composition. Secondary outcomes will be measured by the Weight-Related Symptom Measure, Obesity and Weight Loss Quality of Life, the Power of Food Scale, and the Chinese medicine differential diagnosis questionnaire. Outcomes will be assessed at the baseline, and endpoints of the 3rd, 6th, 9th, 12th, 14th, 16th, and 20th week.

**Discussion:**

This clinical trial will investigate the add-on effect of two combined interventions for weight loss treatment. The findings of this study may contribute to the development of a cost-effective and multidisciplinary weight management approach.

**Trial registration:**

Australia and New Zealand Clinical Trials Registry (ANZCTR) ACTRN12618000964213. Registered on 07 June 2018.

**Supplementary Information:**

The online version contains supplementary material available at 10.1186/s13063-022-06725-8.

## Background

Obesity and overweight are defined as the excessive accumulation of fat in the body which impairs health [1]. Body mass index (BMI) is an indicative tool to estimate the amount of fat deposited in the body. Individuals who have a BMI equal to or above 25 are classified as overweight and obese if their BMI is equal to or above 30 [2].

The worldwide prevalence of overweight and obesity has almost tripled since 1975 [1]. Research has shown that overweight and obesity are linked to various chronic conditions such as cardiovascular diseases, type II diabetes mellitus, hypertension, various types of cancers, gynaecological issues and osteoarthritis [3, 4]. Due to social stigmatization, overweight and obese individuals often suffer from body dissatisfaction, anxiety, depression, lowered self-esteem, and disorders of eating behaviours [5, 6]. These impacts can often lead to increased indirect costs to healthcare and government welfare expenditure and indirect costs such as loss of work productivity [7].

Current weight management includes pharmacotherapy, dietary changes, physical exercise, bariatric surgery and behavioural changes [8, 9]. The cause of overweight and obesity is multifactored, including genetic, physiological, behavioural, sociocultural and psychological influences [10]. Therefore, the Australian clinical practice guidelines for the management of obesity suggest multicomponent lifestyle intervention for weight management, which includes diet changes, increased physical activities and behaviour changes to adopt a healthy lifestyle. Moreover, it is recommended that psychological interventions may improve weight management outcomes [11].

Dietary changes include a low-calorie diet which aims to decrease energy intake to facilitate weight loss [9]. On the other hand, physical exercise increases energy expenditure which can result in weight reduction [12]. However, decreased food intake can cause an increase in hunger sensation and decreased satiety [13]. An increase in the level of physical exercise also induces muscle pain [14]. All these factors may contribute to psychological distress and decrease the tendency to sustain these changes.

Consumption of pharmacological anti-obesity treatment may cause disturbing side effects such as faecal incontinence and deficiency in fat-soluble vitamins [15]. Bariatric surgeries can significantly decrease body weight. However, it may lead to death or complications, such as acid reflux, malnutrition, and bleeding [16, 17].

Acupuncture is considered a type of complementary and alternative medicine [18]. The traditional manual acupuncture treatment involves inserting fine needles into specific acupuncture points on the human body to treat various conditions [19]. Variants of acupuncture have also been developed, such as Electroacupuncture (EA), catgut-embedding and auricular acupuncture [20]. A recent systematic review evaluated the weight management outcome between variants of acupuncture. However, no conclusion has been drawn [20]. EA is one of the variants of acupuncture, which involves connecting two needling sites to an electro-stimulator to allow a predefined intensity of current to pass through the body in a constant pattern. Therefore it is a reproducible stimulation that can be applied in different clinical settings and has gained popularity to be used in research studies [21, 22].

Some studies found that EA reduced more weight than exercise [23, 24] and diet restriction [25]. Guo et al. [26] suggested that EA in combination with diet restriction may produce more weight loss than diet alone [11]. Three other studies have found that EA plus diet and exercise may reduce BMI more than diet and exercise alone [27–29]. Several studies also found that EA significantly reduced weight and BMI in comparison to sham EA [25, 30, 31]. Moreover, Li et al. [32] suggested that acupuncture combined with a diet therapy can be more effective than diet therapy alone. Animal studies indicated that EA can facilitate weight loss through modulating neurotransmitters to suppress appetite [33], promote browning of the white adipose tissue [34] and regulate gut flora [35].

Previous systematic reviews have investigated the effect of acupuncture on improving weight loss in obese people [32, 36–45]. Two systematic reviews suggested that acupuncture can have a positive effect on waist circumference (WC) and hip circumference (HC), reducing the WC by 2.02cm (95%CI 0.21 to 3.83, *p*=0.03) and WC by 2.74cm (95%CI 1.21 to 4.27, *p*=0.0004) [44, 45]. Kim, Trinh [24] reported that acupuncture combined with exercise is more effective than e exercise alone in reducing BMI (Hedges’ *g* = 1.104, 95%CI 0.531 to 1.678) [10]. Chen et al. [46] and our previous research suggested that the mechanism of acupuncture for weight loss may be due to reducing excessive appetite and increasing metabolism [17, 20]. A systematic review on RCT of acupuncture for obesity suggested that acupuncture may lower serum leptin levels in obese adults [47].

Even though there is increasing evidence supporting the use of acupuncture for overweight and obesity [21], the current Australian clinical guidelines for obesity do not include acupuncture as a modality for treatment. Therefore, further investigations are needed to provide more evidence to assist clinical decisions.

Mindfulness meditation (MM) is a psychological approach with increasing popularity for weight management [48]. The term mindfulness is a concept that originated from Buddhist traditions. Mindfulness is defined as the awareness of paying attention to the current moment without judgment to the thought [49]. It has been further defined that mindfulness also involves in sustaining attention through self-regulation [50] and that the current moment should be experienced with an acceptance attitude [51].

A recent systematic review suggested that MM can be effective in improving eating behaviour such as emotional eating and binge eating and can be used in conjunction with other interventions for weight loss treatment [48]. Studies have found that better weight loss outcomes and eating behaviours have been achieved by adding MM to a standard behaviour weight loss program [48, 52].

There is evidence supporting that EA can enhance the outcome of diet and exercise for weight loss [37, 38], and MM can enhance the weight management outcome by improving eating behaviour [48]. However, the combination of EA and MM has never been investigated.

This clinical trial aims to clinically evaluate the add-on effect of MM to EA and vice versa for people with overweight or obesity.

## Methods

### Trial design

**Fig. 1:**
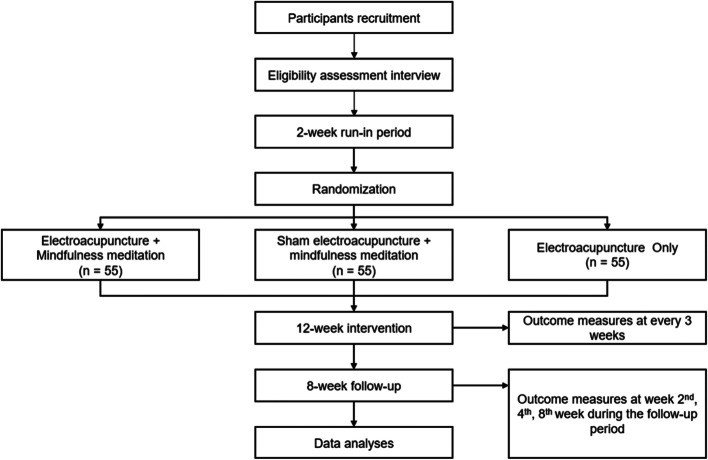
Flow chart for clinical trial procedure

**Fig. 2:**
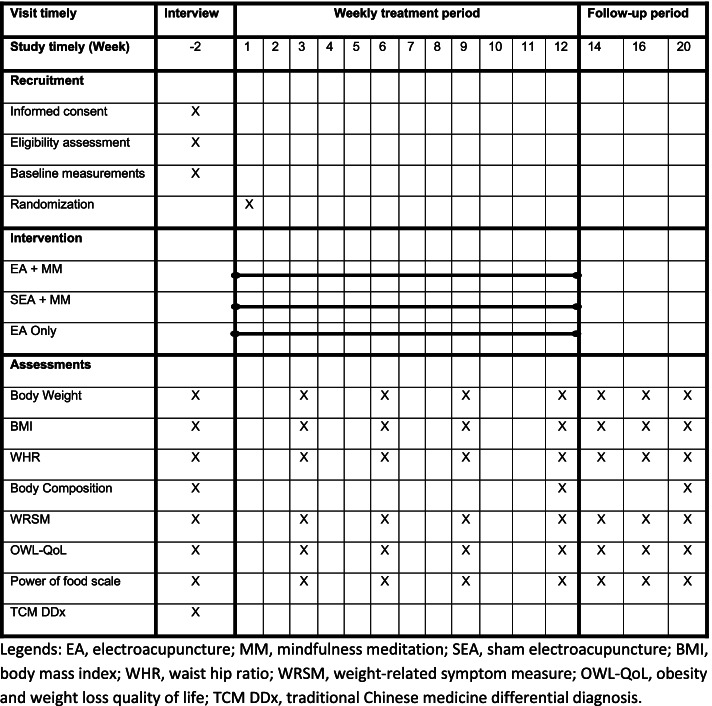
The SPIRIT study schedule

This is a sham-controlled, three-arm randomized superiority clinical trial designed in accordance with the Guideline of National Statement on Ethical Conduct in Human Research 2007 (Updated July 2018) [53]. This clinical trial will be conducted in the Chinese Medicine Research Laboratory at the Royal Melbourne Institute of Technology (RMIT) University Bundoora West and Melbourne city campus. The total duration of this trial will be 22 weeks, which consists of a 2-week run-in period, a 12-week intervention period, and an 8-week follow-up period. Figure 1 shows the flow chart of the clinical trial procedure. The Standard Protocol Items: Recommendations for Interventional Trials (SPIRIT) Checklist and figure are provided in the Additional file 1 and Fig. 2, respectively [54].

### Registration and ethics approval

This study protocol has been developed in accordance with the revised Standards for Reporting Interventions in Clinical Trials of Acupuncture (STRICTA) [55] and the Consolidated Standard of Reporting Trials (CONSORT) guidelines [56]. The RMIT University Human Research Ethics Committee (HERC) has approved this trial protocol with project number 21457. This trial has been registered with the Australian New Zealand Clinical Trial Registry (ACTRN1261800064213).

### Participants

Overweight and obese adult volunteers will be recruited for this study. Participants will be screened according to the following inclusion and exclusion criteria:

### Inclusion criteria

Eligible participants must fulfil the following criteria:

- Being overweight or obese, with BMI between 25 and 39.99 inclusively;
- Adult aged between 18 and 60;
- Be available during the period of this clinical trial; and
- Give written consent to participate in the study

### Exclusion criteria

Participants will be excluded if they have one or more of the following conditions:

- Serious chronic medical conditions, such as cardiovascular diseases, cancer, HIV, and epilepsy;
- Women who are pregnant or lactating;
- Drug-induced secondary obesity;
- Medical conditions which are known to link with obesity, such as uncontrolled high blood pressure, polycystic ovary syndrome, hypothyroidism, Cushing syndrome, and Hashimoto’s disease;
- Mental conditions such as clinical depression, anxiety, PTSD, and psychosis;
- Participants who are not willing to be treated by acupuncture or to practice meditation;
- Difficulties with understanding and reading English;
- Taking blood-thinning medications;
- Auditory impairment who cannot listen to audio records; and
- Participants who received treatment for obesity in the past 3 months, including medical treatment or participated in weight loss programs.

### Sample size

The sample size was calculated based on a previous study comparing the acupuncture group with the sham acupuncture for BMI [57]. After the treatment, the effect size estimate was 0.56. The sample required to achieve 80 per cent power at a significance level of 5% is 55 per group (total *n* = 165).

### Recruitment and screening

All prospective participants will receive a link to two online Microsoft forms, which include the Participant Information and Consent Form (PICF) (Additional file 2) and The General Information and Screening Questionnaires (GISQ). They will be asked to thoroughly read the PICF, and electronically submit the GISQ if they are willing to participate in the trial. The research team will review all GISQ to screen for eligible participants. A screening interview will be scheduled to collect all baseline measurements, ensuring that all participants comply with the selection criteria. Participants who meet the inclusion criteria will be recruited for this clinical trial. The investigators will be responsible for obtaining written consent from the participants before randomization.

### Randomization

Participants will be randomly assigned into three groups. An independent statistician will be responsible for generating a randomization code by using a computer program with a 1:1:1 allocation. The code will be placed into individually sealed opaque envelopes, which contain information about the allocated treatment group. All participants will select their envelope from the available envelopes and hand them over to the researcher. The researcher will ensure that the envelope will not be opened until the name of the allocated participants is written on the envelope. Group assignments will not be changed after the allocation has been made.

### Blinding

The acupuncturist and the researcher who is responsible for playing pre-recorded mindfulness meditation audio records will not be blinded. A research assistant who is responsible for entering outcome measurements is blinded to treatment allocation. The treatment allocation will be blinded to an individual data manager, who is responsible for manually entering information to a securely stored database. To ensure allocation concealment, any discussion between the treatment provider and the participant is required to be kept minimal.

### Interventions

#### Electroacupuncture (EA)

**Fig. 3:**
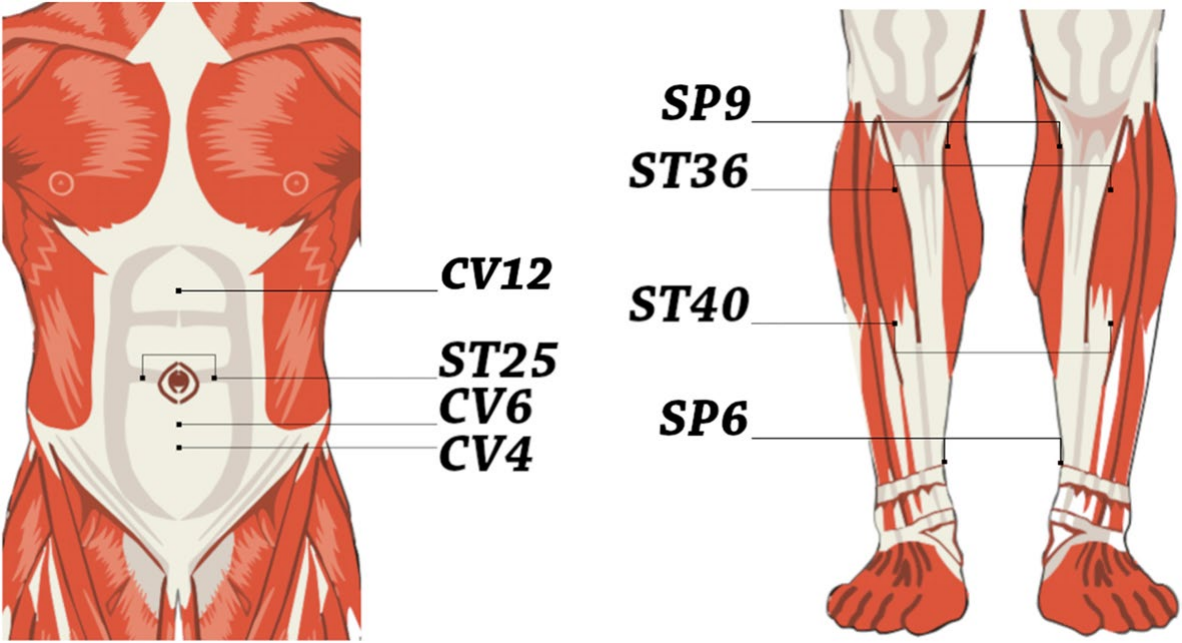
The acupuncture points for EA treatment

The intervention consists of 12 weekly sessions, and each session includes EA treatment with 30 mins needles stimulation. The acupuncture points are Zusanli (ST36), Sanyinjiao (SP6), Tianshu (ST25), Zhongwan (CV12), Fenglong (ST40), Guanyuan (CV4), Qihai (CV6), and Yinlingquan (SP9). The location of the acupuncture points will follow the standard point location in the Western Pacific region published by WHO [58]. Figure 3 below illustrates the location of the acupuncture points for EA treatments.

Sterilized stainless-steel single-use disposable needles (0.25 × 40mm; Seirin, Seirin Corporation, Japan) and electro-stimulator (ES-160; ITO, ITO Company Ltd., Japan) will be in this trial. Skin sterilization will be performed by applying 70% isopropyl alcohol swab to the area. All acupuncture points will be needled perpendicularly with a guide tube. Insertion depth will be approximately 25 mm, depending on the location of individual points. Each point will be connected to the electro-stimulator with fast and slow modes with frequency switching between 33 − 100 Hz at 48 mA peak intensity, and the connections are shown in Table 1. All needles will be removed after 30 min of electro-stimulation.

**Table 1:** Connection of needles to electro-stimulator

Anode (red)	Cathode (black)
CV 12	ST 25 (left)
CV 6	ST 25 (right)
SP 6	SP 9
ST 36	ST40

The acupuncturist who is providing acupuncture treatment in this trial is a registered acupuncturist under AHPRA (Australian Health Practitioner Regulation Agency) as a qualified acupuncturist for more than 3 years of clinical experience.

#### Sham electroacupuncture (SEA)

The SEA also consists of 12 weekly sessions, and this is penetrating acupuncture on non-acupuncture points (1 cm away from the acupuncture point and meridian). The same type of acupuncture needles will be used for SEA with the exact skin sterilization method. All acupuncture points will be needled perpendicularly with a guide tube. Insertion depth will be approximately 25 mm, depending on the location of individual points. Each point will be connected to a sham electro-stimulator (showing a continuous wave at a frequency of 30 Hz but without actual stimulation). All needles will be removed after 30 min of needle retention.

#### Mindfulness meditation

The mindfulness meditation (MM) intervention will involve practising a 10-minute pre-recorded MM during 12 weekly sessions. The MM was adapted from Mindfulness-Based Eating Awareness Training (MB-EAT) developed by Jean Kristeller [59]. It is a sitting meditation to be practised in individual settings, which requires participants to focus on their awareness through breathing.

At the initial session, a copy of the pre-recorded MM audio file will be sent to the ‘participants’ email for daily self-practice. The participants will also receive a copy of the MM information and instruction booklet, which explains the procedure of practising mindfulness meditation. Participants will need to practice daily at home for 10 min, following the instruction by listening to the playback of the MM audio file.

Email reminders will be sent to the participants for appointments to improve adherence to intervention protocols. A log book will be provided to record the frequency and duration of each MM self-practice.

### Group design

This is a three-arm clinical trial, which consists of the EA plus MM group (EAM), SEA plus MM group (SAM) and EA only group (EAO). Participants will be asked to continue with their usual diet and exercise, and refrain from participating in any weight loss program or consuming any weight loss supplements/medications, otherwise will be considered as a dropout during the study. The description of each group is listed below (Table 2):

**Table 2:** Group description

Group	Description
Electroacupuncture + MM (EAM)	This group consists of a combination of EA and mindfulness meditation interventions. Participants will be given the abovementioned EA treatment. They will then be prompted to follow the 10 min pre-recorded MM instruction. They will be instructed to practice MM daily.
Sham electroacupuncture + MM (SAM)	This group consists of a combination of EA and mindfulness meditation interventions. Participants will be given the abovementioned sham EA treatment. They will then be prompted to follow the 10 min pre-recorded MM instruction. They will be instructed to practice MM daily.
Electroacupuncture only (EAO)	This group consists of the abovementioned electroacupuncture treatment only.

*Abbreviations*: *MM* Mindfulness meditation, *EA* Electro-acupuncture

The add-on effect of MM to EA comparing the treatment group (EAM) with the control group (SAM). On the other hand, to assess the add-on effect of electroacupuncture on mindfulness meditation, the treatment group (EAM) will be compared with the control group (EAO).

Participants will confirm their next appointment during their visits, and a message will be sent to the participants to remind and confirm their weekly visits.

## Outcome measurements

### Primary outcome

#### Weight and body mass index (BMI)

The primary focus of the outcome measurements is on the effectiveness of the interventions in reducing the severity of overweight or obesity. It includes the change in body weight in Kg and BMI. BMI is a commonly used tool for assessing the risk of overweight and obesity for individuals [1]. It is calculated using the following equation:

$$\mathrm{BMI}=\frac{\mathrm{Mass}\ \left(\mathrm{Kg}\right)}{\left(\mathrm{Height}\ \left(\mathrm{m}\right)\right){\mathrm{H}}^2}$$

#### Waist and hip ratio (WHR)

WHR is another commonly used tool to measure central obesity, which will be calculated as:

$$\mathrm{WHR}=\frac{\mathrm{Waist}\kern0.5em \mathrm{circumference}\kern0.5em \left(\mathrm{cm}\right)}{\mathrm{Hip}\kern0.5em \mathrm{circumference}\kern0.5em \left(\mathrm{cm}\right)}$$

Participants will be required to wear similar types of clothing to avoid the influence of clothing on the measured weight and the circumferences.

#### Body composition

Body composition involves measuring the body fat percentage (Fat %) by using the Dual Energy X-ray Absorptiometry (DXA) scan. DXA is a type of medical imaging technology which uses low dose radiation for the whole-body scan to provide the measurement of Fat %. The scanning will be performed by qualified operators.

The measurements of body composition will be taken from the baseline, the 12th and 20th-week visit.

### Secondary outcomes

#### Weight-Related Symptom Measure (WRSM) and Obesity and Weight Loss Quality of Life (OWL-QOL)

WRSM and OWL-QOL are two qualitative instruments to evaluate the weight-related quality of life and symptoms. WRSM is a 20-item questionnaire that evaluates the weight-related symptoms such as tiredness and increased appetite. The responses will be recorded on a 0 to 6 points scale, where 0 indicates not at all and 6 indicate a very great deal. The higher the total score indicates more weight-related symptoms which affect the ‘participants’ well-being. The OWL-QOL is a 17-item questionnaire that measures ‘participants’ weight-related feelings. Participants will respond to questions similar to ‘Because of my weight, I try to wear clothes that hide my shape’ or ‘I feel ugly because of my weight’. The responses will be recorded on a 0–6-point scale, where 0 indicates not at all and 6 indicate a very great deal. The higher the total score indicates the quality of life is being affected by obesity.

#### Power of Food Scale

Power of Food Scale (PSF) is a 15-item scale which measures psychological influence on food intake, particularly in an environment where palatable food is highly abundant. Participants will respond to questions like ‘I think I enjoy food more than many other people or I cannot help myself thinking about food’. The responses will be recorded in a 1- to 5-point scale, where 1 indicates disagree and 5 indicates strongly agree. The higher the total score indicates a higher food intake due to psychological influence.

#### Frequency of practising MM

The self-practice MM will also be recorded to assess the correlation between the frequencies of practice with other measured outcomes. A log book will be given to participants who are allocated to EAM and SAM groups. Participants will have an option to submit a photo of their log book via email or to hand in the hard copy.

#### Chinese medicine differential diagnosis questionnaire

The Chinese medicine differential diagnosis questionnaire will be used during the baseline measurement. The questionnaire will differentiate participants' symptoms into 5 different patterns according to Chinese medicine theory. The patterns will be used for data analysis on whether EA for weight management is in favour of any type of Chinese medicine differential pattern.

Except for body composition, the abovementioned outcome measurements will be assessed at the endpoints of 3, 6, 9, 12, 14,16, and 20th week.

### Adverse events

All adverse events will be documented, including the description, severity, duration, and the required treatment. All cases of serious adverse events will be immediately reported to the chief investigator for appropriate action. All severe adverse events will be reported to RMIT Human Research Ethics Committee (HREC), and the RMIT HREC will make the final decision on the termination of the trial.

### Attendance and drop-out

Participants will be informed that they are free to withdraw from the clinical trials at any time. The attendance to the treatment will be recorded for participant adherence. The reason for withdrawal will also be followed up and documented. Participants are not obligated to provide a reason for withdrawal. However, if the reason is related to adverse events, they will be follow-up until the issue is completely resolved. Participants who undergo bariatric surgery, consume weight loss medication, or participate in another weight loss program during this study will be considered drop-out.

### Data management

The personal information and records of each participant will be kept in an individual file and stored in a locked cabinet. All electronic documents will be stored in an RMIT University computer with password protection. Access to all research data will only be permitted to an authorized research team member.

All participants will receive a summary of study findings and group information upon trial completion. All personal identities will be removed before the publication of the trial findings.

### Statistical analysis

Statistical analysis will be performed using Statistical Package for Social Science (SPSS) at the Discipline of Chinese Medicine, School of Health and Biomedical Science, RMIT University, at the end of the trial. Given the short duration of the trial, no interim analysis will be conducted. Intention-to-treat (ITT) analysis will be used for participants who are randomized and participated for at least one treatment session. The Worst-Case Scenario method will be employed to deal with the missing data. Continuous variables will be summarised using the mean, standard deviation, and 95% confidence intervals, while quantitative variables will be presented using the maximum and minimum values. Baseline data and clinical characteristics will be used to compare the baseline data between intervention groups. Analysis of covariance (ANCOVA) will be performed on BMI and WHR to determine the mean difference between groups at the end of treatment and follow-up period. All comparisons will be two-tailed, and *p* values < 0.05 will be considered statistically significant.

## Discussion

Overweight and obesity have reached an epidemic level across the world over the past decades [1]. More effective weight management strategies are urgently needed to reduce the prevalence and impacts on our society. Due to the multifactorial cause of overweight and obesity, interventions that only target one single factor may not achieve a satisfying weight management outcome [60–62].

Evidence has shown that acupuncture is effective in weight management when it is being used alone or combined with other weight loss interviews to enhance weight loss outcomes [32, 38, 40]. EA is a variant of acupuncture with a replicable mode of stimulation that enables consistent stimulation throughout treatment sessions [21]. Additionally, other evidence has suggested that mindfulness meditation may be added to weight loss programs to enhance the treatment results [52]. Furthermore, the clinical practice guidelines for the management of overweight and obesity in adults, adolescents and children in Australia suggest that psychological component can be added to weight loss programs to increase the likelihood of success [11]. To our best knowledge, this is the first trial protocol evaluating the effect and safety of EA combined with mindfulness meditation for weight management.

The aim of this RCT is to evaluate the add-on effect of EA and MM on weight loss. In terms of group design, the add-on effect of EA will be evaluated by comparing the EAM group and SAM group. The add-on effect of MM will be assessed by comparing the EAM group and the EAO group. This design has put a limitation on this trial in which we cannot blind the participants for the EAO group who will not be participating in the MM component. Participants in the EAM and SAM groups will be blinded by sham electroacupuncture treatment. The SA method was designed as penetrating insertion to a non-acupuncture point, all acupuncturists will receive training on needling to the predefined non-acupuncture points to ensure consistency of point location for the SAM group. The wire of the electro-stimulator will be modified to stop the current being flow to the body; this ensures that the SAM group participants receive no electro-stimulations at all. Participants will be told that the intensity of stimulation is predefined, and therefore they may or may not be able to feel for the stimulation.

Participants in EAM and SAM groups are required to practice MM daily and record their practice in a log book. The pre-recorded MM instructions will increase the flexibility for participants to practice MM at their preferable time.

Both objective and subjective outcome measurements will be evaluated in this trial. The current management of overweight and obesity includes lifestyle changes, pharmacotherapies and surgeries [63], and it is necessary to isolate the effect of these approaches to impact the study results. Therefore, participants will be asked not to undergo bariatric surgery or consume weight loss medications and continue their usual diet and physical activities. As a result, we can ensure that the change in the treatment outcome was attributed to the effect of the allocation study group. Although BMI is the major tool for assessing weight status [1], body fat composition will also be evaluated because it is necessary to investigate whether the reduction in BMI is due to the loss of body fat, muscle mass or bone density [64, 65]. Moreover, WHR is a commonly used tool for assessing abdominal obesity, linked closely to cardiovascular disease risk [66]. The idea of addressing overweight and obesity is to reduce the risk for other comorbidities like cardiovascular diseases [67]. Therefore, WHR will also be assessed to evaluate the effect of this combined intervention on abdominal obesity [68].

The WRSM, OWL-QoL, and Power of Food Scale were used to assess the psychological impact on overweight and obesity [69, 70]. If the result of this RCT is found positive, it will provide a novel cost-effect multicomponent weight management approach. It is anticipated that this approach may improve both physical and psychological outcomes for overweight and obese individuals.

Finally, this rigorously designed clinical trial protocol was developed by following the SPIRIT [54], CONSORT [56], and STRICTA [55] guidelines to minimize the risk of bias and the quality assurance for the combined intervention. If successful, this proposed protocol may provide a safe and cost-effective multidisciplinary approach for weight management.

## Trial status

This is protocol version 2.0. The advertisement for the recruitment commenced in May 2019 after the ethics approval by the RMIT Human Research Ethics Committee (HREC). It is anticipated that the recruitment will be completed by the end of June 2023. The recruitment is currently in progress.

## Supplementary Information

**Additional file 1.**

**Additional file 2.** Participant Information Sheet/Consent Form.

## Data Availability

Not applicable. This paper is a protocol for a randomized clinical trial and does not contain any data.

## Abbreviations

ANCOVA: Analysis of covariance
BMI: Body mass index
DXA: Dual-energy X-ray absorptiometry
EA: Electroacupuncture
EAM: Electroacupuncture plus mindfulness meditation
EAO: Electroacupuncture only
F%: Body fat percentage
HREC: Human Research Ethics Committee
ITT: Intention-to-treat
MB-EAT: Mindfulness-Based Eating Awareness Training
MM: Mindfulness meditation
OWL-QoL: Obesity and Weight Loss Quality of Life
PSF: Power of Food Scale
PTSD: Post-traumatic stress disorder
RMIT: Royal Melbourne Institute of Technology
SAM: Sham acupuncture plus mindfulness meditation
SPSS: Statistical Package for Social Science
TCM: Traditional Chinese medicine
WHO: World Health Organization
WHR: Waist and hip ratio
WRSM: Weight-Related Symptom Measure

## References

[1] World Health Organization. Obesity and overweight fact sheets. Geneva; 2020.

[2] Yumuk V, Tsigos C, Fried M, Schindler K, Busetto L, Micic D (2015). European guidelines for obesity management in adults. Obes Facts..

[3] Williams EP, Mesidor M, Winters K, Dubbert PM, Wyatt SB (2015). Overweight and obesity: prevalence, consequences, and causes of a growing public health problem. Curr Obes Rep.

[4] Kopelman P (2000). Obesity as a medical problem. Nature.

[5] McCuen-Wurst C, Ruggieri M, Allison KC (2018). Disordered eating and obesity: associations between binge eating-disorder, night-eating syndrome, and weight-related co-morbidities. Ann N Y Acad Sci.

[6] Flint S (2015). Obesity stigma: Prevalence and impact in healthcare. Br J Obesity.

[7] Tremmel M, Gerdtham U, Nilsson PM, Saha S (2017). Economic burden of obesity: a systematic literature review. Int J Environ Res Public Health.

[8] Bray GA, Heisel WE, Afshin A, Jensen MD, Dietz WH, Long M (2018). The science of obesity management: an endocrine society scientific statement. Endocr Rev.

[9] Rajmil L, Bel J, Clofent R, Cabezas C, Castell C, Espallargues M (2017). Clinical interventions in overweight and obesity: a systematic literature review 2009–2014. An Pediatr (Engl Ed).

[10] Uzogara S (2017). Obesity epidemic, medical and quality of life consequences: a review. Int J Public Health Res.

[11] National Health and Medical Research Council (2013). Clinical practice guidelines for the management of overweight and obesity in adults, adolescents and children in Australia.

[12] Petridou A, Siopi A, Mougios V (2019). Exercise in the management of obesity. Metabolism.

[13] Drapeau V, Jacob R, Panahi S, Tremblay A (2019). Effect of energy restriction on eating behavior traits and psychobehavioral factors in the low satiety phenotype. Nutrients.

[14] Hulens M, Vansant G, Lysens R, Claessens AL, Muls E (2001). Exercise capacity in lean versus obese women. Scand J Med Sci Sports.

[15] Singh AK, Singh R (2020). Pharmacotherapy in obesity: a systematic review and meta-analysis of randomized controlled trials of anti-obesity drugs. Expert Rev Clin Pharmacol.

[16] Neovius M, Bruze G, Jacobson P, Sjöholm K, Johansson K, Granath F (2018). Risk of suicide and non-fatal self-harm after bariatric surgery: results from two matched cohort studies. Lancet Diabetes Endocrinol.

[17] Chang SH, Freeman N, Lee J, Stoll C, Calhoun A, Eagon J (2018). Early major complications after bariatric surgery in the USA, 2003–2014: a systematic review and meta-analysis. Obes Rev.

[18] Bertisch SM, Wee CC, McCarthy EP (2008). Use of complementary and alternative therapies by overweight and obese adults. Obesity.

[19] Chae Y, Olausson H (2017). The role of touch in acupuncture treatment. Acupunct Med.

[20] Zhang Y, Li J, Mo G, Liu J, Yang H, Chen X (2018). Acupuncture and related therapies for obesity: a network meta-analysis. Evid Based Complement Alternat Med.

[21] Mayor D (2013). An exploratory review of the electroacupuncture literature: clinical applications and endorphin mechanisms. Acupunct Med.

[22] Napadow V, Makris N, Liu J, Kettner NW, Kwong KK, Hui KK (2005). Effects of electroacupuncture versus manual acupuncture on the human brain as measured by fMRI. Hum Brain Mapp.

[23] Hsu C-H, Hwang K-C, Chao C-L, Lin J-G, Kao S-T, Chou P (2005). Effects of electroacupuncture in reducing weight and waist circumference in obese women: a randomized crossover trial. Int J Obes.

[24] Kim J, Trinh KV, Krawczyk J, Ho E (2016). Acupuncture for obesity: a systematic review. J Acupunct Tuina Sci.

[25] Cabioglu MT, Ergene N, Tan Ü (2007). Electroacupuncture treatment of obesity with psychological symptoms. Int J Neurosci.

[26] Guo Y, Xing M, Sun W, Yuan X, Dai H, Ding H (2014). Plasma nesfatin-1 level in obese patients after acupuncture: a randomised controlled trial. Acupunct Med.

[27] Yang JJ, Xing HJ, Wang SJ, Xiao HL, Li M (2010). The influence of BMI and serum lipid on simple obesity patients by using the treatment of acupuncture plus diet and exercise. Shizhen Guo Yi Guo Yao.

[28] Gao H, Xing HJ, Yang JJ, Xiao HL (2010). The influence of BMI on simple obesity patients by acupuncture treatment with diet changes and aerobic exercise. J Hebei TCM Pharmacol.

[29] Zhao LQ, Shi Y (2010). Clinical investiagtion on Electroacupuncture with diet and exercise in treating stomach and intesting excessive heat type of simple obesity. J An Hui TCM College.

[30] Zhao Y, Liu J, Liu Y, Lin MQ (2011). Clinical research of using acupuncture, which can invigorate spleen and eliminate phlegm, to treat simple obesity. J Sichuan Trad Chinese Med.

[31] Zhang L, Zhou XL, Zhang HM, Hu MQ (2012). Observation on clinical effectiveness of electroacupuncture on simple obesity. J Sichuan Trad Chinese Med.

[32] Li KX, Yang AW, Xue CC, Lenon GB (2015). Traditional Chinese manual acupuncture for management of obesity: A systematic review. World J Metaanal.

[33] Wang F, Tian D-R, Han J-S (2008). Electroacupuncture in the Treatment of Obesity. Neurochem Res.

[34] Wang L, Li J, Huang W, Ran G, Zhang Y, Zhuo Y (2018). Effect of electroacupuncture on the expression of PGC-1α and UCP-1 in the brown adipose tissue of obese rats. World J Acupunct Moxibustion.

[35] Si Y-C, Miao W-N, He J-Y, Chen L, Wang Y-L, Ding W-J (2018). Regulating gut flora dysbiosis in obese mice by electroacupuncture. Am J Chinese Med.

[36] Chen L, Fang J, Jin X, Keeler CL, Gao H, Fang Z (2016). Acupuncture treatment for ischaemic stroke in young adults: protocol for a randomised, sham-controlled clinical trial. BMJ Open.

[37] Cho S, Lee J, Thabane L, Lee J (2009). Acupuncture for obesity: a systematic review and meta-analysis. Int J Obes.

[38] Fang S, Wang M, Zheng Y, Zhou S, Ji G (2017). Acupuncture and lifestyle modification treatment for obesity: a meta-analysis. Am J Chinese Med.

[39] Kim S, Shin I, Park Y (2018). Effect of acupuncture and intervention types on weight loss: a systematic review and meta-analysis. Obes Rev.

[40] Li X (2015). To evaluate the abdominal acupuncture treatment for simple obesity by systematic review [master's thesis].

[41] Lin X, Li B, Du Y, Xiong J, Sun P (2009). Systematic evaluation of therapeutic effect of acupuncture for treatment of simple obesity. Zhongguo Zhen Jiu.

[42] Liu Q (2014). Acupuncture for simple obesity: a systematic review [master's thesis].

[43] Xia Y (2015). Acupuncture for obesity: a systematic review [master's thesis].

[44] Zhang RQ, Han LX, Tan J, Xin B, Liu QL, Sun N (2017). Meta-analysis of randomized clinical trials about acupuncture treatment for female simple obesity. Zhiye Yu Jiankang.

[45] Zhang R-Q, Tan J, Li F-Y, Ma Y-H, Han L-X, Yang X-L (2017). Acupuncture for the treatment of obesity in adults: a systematic review and meta-analysis. Postgrad Med J.

[46] Chen X, Huang W, Deng J, Cheng XL, Chen L, Zhou ZY (2016). Effect of Acupuncture Therapy on Simple Obesity in Adults : A Meta-Analysis. J Clin Acupunct Moxibustion.

[47] Park KS, Park KI, Suh HS, Hwang DS, Jang JB, Lee JM (2017). The efficacy and safety of acupuncture on serum leptin levels in obese patients: a systematic review and meta-analysis. Eur J Integr Med.

[48] Katterman SN, Kleinman BM, Hood MM, Nackers LM, Corsica JA (2014). Mindfulness meditation as an intervention for binge eating, emotional eating, and weight loss: a systematic review. Eat Behav.

[49] Kabat-Zinn J (2003). Mindfulness-based interventions in context: past, present, and future. Clin Psychol (New York).

[50] Bishop SR (2004). Mindfulness: A Proposed Operational Definition. Clin Psychol (New York).

[51] Shapiro SL, Carlson LE, Astin JA, Freedman B (2006). Mechanisms of mindfulness. J Clin Psychol.

[52] Spadaro KC, Davis K, K., Sereika Susan M, Gibbs Bethany B, Jakicic John M, Cohen Susan M. Effect of mindfulness meditation on short-term weight loss and eating behaviors in overweight and obese adults: a randomized controlled trial. JCIM. 2017;5(2) Available from: https://www.degruyter.com/view/j/jcim.ahead-of-print/jcim-2016-0048/jcim-2016-0048.xml. 10.1515/jcim-2016-0048 29211681

[53] The National Health and Medical Research Council (2007). National Statement on Ethical Conduct in Human Research 2007 (Updated 2018).

[54] Chan A-W, Tetzlaff JM, Altman DG, Laupacis A, Gøtzsche PC, Krleža-Jerić K (2013). SPIRIT 2013 Statement: Defining Standard Protocol Items for Clinical Trials. Ann Int Med.

[55] MacPherson H, Altman DG, Hammerschlag R, Youping L, Taixiang W, White A, et al. Revised Standards for Reporting Interventions in Clinical Trials of Acupuncture (STRICTA): Extending the CONSORT Statement. J Altern Complement Med. 2010;7(6). 10.1089/acm.2010.1610 20954957

[56] Schulz KF, Altman DG, Moher D. For the CG. CONSORT 2010 statement: updated guidelines for reporting parallel group randomised trials. BMC Med. 2010:8–18. 10.1186/1741-7015-8-18 PMC2860339 20334633

[57] Güçel F, Bahar B, Demirtas C, Mit S, Çevik C (2012). Influence of acupuncture on leptin, ghrelin, insulin and cholecystokinin in obese women: a randomised, sham-controlled preliminary trial. Acupunct Med..

[58] World Health Organization (2008). WHO standard acupuncture point locations in the Western Pacific Region.

[59] Kristeller JL, Hallett CB (1999). An exploratory study of a meditation-based intervention for binge eating disorder. J Health Psychol.

[60] Wang Z, Yuan D, Duan Y, Li S, Hou S (2017). Key factors involved in obesity development. Eat Weight Disord.

[61] Heymsfield SB, Wadden TA (2017). Mechanisms, pathophysiology, and management of obesity. N Engl J Med.

[62] Kyrou I, Chrousos GP, Tsigos C (2006). Stress, visceral obesity, and metabolic complications. Ann N Y Acad Sci.

[63] Bray GA, Frühbeck G, Ryan DH, Wilding JPH (2016). Management of obesity. The Lancet.

[64] Hunter GR, Bryan DR, Borges JH, David Diggs M, Carter SJ (2018). Racial differences in relative skeletal muscle mass loss during diet-induced weight loss in women. Obesity.

[65] Shapses SA, Sukumar D (2012). Bone metabolism in obesity and weight loss. Annu Rev Nutr.

[66] De Koning L, Merchant AT, Pogue J, Anand SS (2007). Waist circumference and waist-to-hip ratio as predictors of cardiovascular events: meta-regression analysis of prospective studies. Eur Heart J.

[67] Bischoff SC, Schweinlin A (2020). Obesity therapy. Clin Nutr ESPEN.

[68] Lam BCC, Koh GCH, Chen C, Wong MTK, Fallows SJ (2015). Comparison of body mass index (BMI), body adiposity index (BAI), waist circumference (WC), waist-to-hip ratio (WHR) and waist-to-height ratio (WHtR) as predictors of cardiovascular disease risk factors in an adult population in Singapore. PLoS One.

[69] Niero M, Martin M, Finger T, Lucas R, Mear I, Wild D (2002). A new approach to multicultural item generation in the development of two obesity-specific measures: the obesity and weight loss quality of life (OWLQOL) questionnaire and the weight-related symptom measure (WRSM). Clin Ther.

[70] Lowe MR, Butryn ML, Didie ER, Annunziato RA, Thomas JG, Crerand CE (2009). The power of food scale. a new measure of the psychological influence of the food environment. Appetite.

