# Correction: The clinical evaluation of electroacupuncture combined with mindfulness meditation for overweight and obesity: study protocol for a randomized sham-controlled clinical trial

**DOI:** 10.1186/s13063-023-07156-9

**Published:** 2023-03-21

**Authors:** Ching Yee Chung, Angela Wei Hong Yang, Alexander De Foe, Mingdi Li, George Binh Lenon

**Affiliations:** 1 Discipline of Chinese Medicine, School of Health and Biomedical Sciences, RMIT University, PO Box 71, Bundoora, VIC 3083 Australia; 2 School of Educational Psychology and Counselling, Faculty of Education, Monash University, Clayton, Australia; 3 Department of Preventative and Health Care, Qingdao Traditional Chinese Medicine Hospital (Qingdao Hiser Hospital), Qingdao, Shandong China

**Correction: Trials 23, 818 (2022)**

**https://doi.org/10.1186/s13063-022-06725-8**

The original publication of this article [1] contained 2 errors:

1. The author name “Foe” should be “De Foe”

2. The affiliation of “Alexander De Foe” should be “School of Educational Psychology and Counselling, Faculty of Education, Monash University, Clayton, Australia”

The original publication has been updated.
