# Supplementary material for: The clinical evaluation of electroacupuncture combined with mindfulness meditation for overweight and obesity: study protocol for a randomized sham-controlled clinical trial
Source: Trials. 2022 Sep 27;23:818. doi: 10.1186/s13063-022-06725-8 (PMC9513962; doi:10.1186/s13063-022-06725-8)
Supplement: Supplementary file 2 — Additional file 2. Participant Information Sheet/Consent Form. [file 13063_2022_6725_MOESM2_ESM.docx]

**
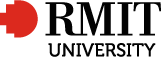
**

**Participant Information Sheet/Consent Form**

| **Title** | The clinical evaluation of electroacupuncture combined with mindfulness meditation in the weight management |
| --- | --- |
| **Protocol Number** | TBA |
| **Chief Investigator/Senior Supervisor** | Dr. George Lenon |
| **Associate Investigator(s)/Associate Supervisor(s)** | Dr. Angela Wei Hong Yang  Dr. Alexander De Foe |
| **Principal Research Student(s)** | Ms. Ching Yee Chung |
| **Location** | Melbourne, Australia |

**What does my participation involve?**

**1 Introduction**

You are invited to take part in this research project, which is called the clinical evaluation of Electroacupuncture combined with mindfulness meditation in weight management. You have been invited because you have this condition and may meet the inclusion criteria for this clinical trial.

This Participant Information Sheet/Consent Form tells you about the research project. It explains the processes involved with taking part. Knowing what is involved will help you decide if you want to take part in the research.

Please read this information carefully. Ask questions about anything that you don’t understand or want to know more about. Before deciding whether or not to take part, you might want to talk about it with a relative or friend.

Participation in this research is voluntary. If you don’t wish to take part, you don’t have to.

If you decide you want to take part in the research project, you will be asked to sign the consent section. By signing it you are telling us that you:

• Understand what you have read

• Consent to take part in the research project

• Consent to be involved in the research described

• Consent to the use of your personal and health information as described.

You will be given a copy of this Participant Information and Consent Form to keep.

**2 What is the purpose of this research?**

The prevalence of obesity has almost been tripled in the past 40 years. Acupuncture has been used for treating various conditions for more than 3000 years. Mindfulness meditation (MM), on the other hand, is a relatively new psychotherapy which involves practising meditation to treat various conditions. The current Australian clinical guidelines for obesity suggest a multidisciplinary approach for treating obesity, and adding psychological intervention may enhance the outcome of weight loss programs. Acupuncture treatments in Australia are not covered by the national Medicare scheme and patients who seek acupuncture treatment will incur out-of-pocket expenses.

Electroacupuncture is a variant of acupuncture, which involves connecting the body acupuncture needles with an electro-stimulator to induce a re-producible, continuous stimulation to the needling site. Our research team in the discipline of Chinese Medicine at RMIT University has already performed extensive research in relation to obesity. We would like to investigate the combined effect of electroacupuncture and mindfulness meditation in weight management to assist weight loss, improve eating behaviours and quality of life during the weight loss process.

The results of this research will be used by the researcher, Ms Ching Yee Chung, to obtain a Doctor of Philosophy degree in complementary medicine.

This research has been initiated by the researcher, Dr George Binh Lenon, Discipline of Chinese Medicine, RMIT University.

**3 What does participation in this research involve?**

If you would like to participate in this research project, you must be between 18 to 60 years old and your condition must meet the inclusion criteria of this study, which includes having BMI between 25 – 39.99 kg/m^2^. You must not have any obesity treatment within the past three months, which includes medical treatment or weight loss programs. You will not be able to participate if you have cardiovascular diseases, uncontrolled high blood pressure, polycystic ovary syndrome, hypothyroidism, Cushing syndrome, cancer, HIV, epilepsy, Hashimoto’s Disease, lactating, depression, anxiety, post-traumatic stress disorder (PTSD), taking blood thinning medications. You will also not be able to participate if you are planning pregnancy, pregnant, planning to travel during the period of the clinical trial, practising Chinese Medicine, or have any relationship to the Discipline of Chinese Medicine, including staff, past and current students. To minimise potential risks for adverse events, it will be of benefit to fully disclose any known current or previous medical conditions to the research team.

You would need to complete two questionnaires online for assessing your eligibility to this research project. The time required for completing the questionnaires is approximately 20-30 minutes.

The result of the screening process will be emailed to you within two weeks of your screening questionnaires submission. The result will indicate whether you are potentially eligible for taking part in this research project. If the screening questionnaire shows that you cannot be in the research project, the research coordinator will discuss other options with you.

If you are potentially eligible for this research project, you will then be invited to attend an assessment interview at the Clinical Trial Laboratory at RMIT University West campus. You are encouraged to ask any questions in relation to your concerns before making the decision to participate in this research study. You will need to sign an informed consent form if you are willing to proceed with the participation. To confirm your eligibility, body measurements of weight and height will be taken to ensure that your BMI meet the inclusion criteria. You will be excluded from this trial if your BMI does not fall between 25-39.99. It is important to aware that you are still free to withdraw from this study after signing the informed consent form.

Upon information consent, you will be assessed by taking body measurements by our research team, which includes waist circumference and hip circumference. Your fat composition will be measured by using the by using the Dual Energy X-ray Absorptiometry (DEXA) scan.

You will also need to fill out assessment questionnaires (Power of food scale, weight-related symptoms measure, obesity and weight loss quality of life), and undertake a Chinese Medicine differential diagnosis. You will also be asked to randomly select one envelope which determines your treatment group. You will have 1 in 3 chances to be randomised to receive real electroacupuncture and mindfulness meditation treatment, or to receive sham electroacupuncture and mindfulness meditation treatment, or to receive real electroacupuncture treatment only. Sham electroacupuncture is puncture on non-acupuncture point without real electrostimulation, which is not supposed to have therapeutic effects. This is a type of control which is used for comparing the effect of the real electroacupuncture treatment.

You may be treated by a practitioner of your opposite gender. You will need to inform the research team if you wish to be treated by a practitioner of the same gender.

The participation of this research project includes the initial assessment interview, a 2 week run-in period, 12 weeks of treatment period and an 8 week follow-up period. Therefore, you will be involved in this study for a total of 22 weeks continuously.

You will need to attend to the Clinical Trial Laboratory weekly during the 12 week treatment period, and also three follow up visits on 14^th^,16^th^ and 20^th^ week.

You will receive acupuncture treatment during your weekly treatment period. You will be needled on 8 acupuncture points and the needles will then be connected to an electro-stimulator. Needle retention and electro-stimulation in each session will be 30 minutes. It is expected that the each acupuncture treatment will take approximately 45 minutes to 1 hour. If you have been randomised into treatment groups that incorporate mindfulness meditation, you will be staying in the treatment room after the acupuncture treatment and self-practising mindfulness meditation by following a 10 minute pre-recorded audio instruction. You will be given a copy of the mindfulness meditation information and instruction booklet in the first treatment session. The booklet explains what mindfulness meditation is, and provides clear instruction for practicing mindfulness meditation. You will need to self-practise mindfulness meditation daily in your own time and be given a logbook to record the frequency and duration of your self-practised mindfulness meditation.

Body measurements will be taken during your visit at the end of the 3^rd^, 6^th^, 9^th^, 12^th^, 14^th^, 16^th^ and 20^th^ week. You will also be asked to fill out the assessment forms after taking the body measurements. You may also fill out an adverse events form, in case of any unexpected body sensations occur in relation to the treatments. These measurements will take approximately 15 minutes to be completed. A separate will be schedule for your body composition scan, which will be taken during the 12^th^ and 20^th^ week. All the recorded data will be kept in your participant file and securely stored in a locked cabinet in the research room.

This research project has been designed to make sure the researchers interpret the results in a fair and appropriate way and avoids researchers or participants jumping to conclusions.

There are no costs associated with participating in this research project, nor will you be paid.

**4 Other relevant information about the research project**

This is a randomised, sham controlled clinical trial research project. There will be approximately 165 participants in this study from the general population from Melbourne. You will have one-third of a chance to be randomised into one of these three groups:

- Electroacupuncture plus mindfulness meditation group
- Sham electroacupuncture plus mindfulness meditation group
- Electroacupuncture group

The treatment groups are designed for evaluate acupuncture combined with mindfulness meditation in weight management. Electroacupuncture treatment involves needling to acupuncture points; the needling site will be connected to an electrostimulator which provides a constant stimulation to the acupuncture point. The sensation experienced should be strong but not painful. The sham electroacupuncture group will be needled to safe but non-acupuncture points. It is necessary to have a sham electroacupuncture group as a control group, so that the effectiveness of electroacupuncture in weight management can be evaluated. It is also necessary to have an electroacupuncture only group in order to compare with the add-on effect of mindfulness meditation to electroacupuncture.

**5 Do I have to take part in this research project?**

Participation in any research project is voluntary. If you do not wish to take part, you do not have to. If you decide to take part and later change your mind, you are free to withdraw from the project at any stage.

If you do decide to take part, you will be given this Participant Information and Consent Form to sign and you will be given a copy to keep.

Your decision whether to take part or not to take part, or to take part and then withdraw, will not affect your relationship with the researchers or with RMIT University.

**6 What are the possible benefits of taking part?**

We cannot guarantee or promise that you will receive any benefits from this research; however, you may appreciate contributing to knowledge advancement. You may also benefit from improving generally well-being by practicing mindfulness meditation. If the study has significant outcomes, you will benefit for improving your weight management. You may choose to continue with practicing mindfulness meditation, which maybe a more cost effective way to improve your weight management in the long run.

**7 What are the risks and disadvantages of taking part?**

The acupuncture points selected in the study have been used in various types of study and no serious adverse event has been documented. You may feel a prick sensation during insertion of the needles. The most commonly experienced sensation after needle insertion would be numbness, soreness or dullness around the site of insertion. There is no evidence of a serious adverse event being reported for practising a brief mindfulness meditation; however, in case of potential serious psychological disturbances being experienced, the chief investigator will be notified and you may be referred to our team counsellor if necessary. You will also be able to document any adverse event in an adverse event form such as pain reaction or bruising.

You may feel that some of the questions we ask are stressful or upsetting. If you do not wish to answer a question, you may skip it and go to the next question, or you may stop immediately. If you become upset or distressed as a result of your participation in the research project, you may contact our team counsellor directly or notify our investigator so that the research team will be able to arrange for counselling or other appropriate support. Any counselling or support will be provided by qualified staff who are not members of the research team. This counselling will be provided free of charge.

This research project involves exposure to a very small amount of radiation by undertaking DEXA scan. DEXA scan is a type of advanced X-ray medical imaging technology, which involves in using ionising radiation to measure body composition, including fat mass and bone density. As part of everyday living, everyone is exposed to naturally occurring background radiation and receives a dose of about 2 millisieverts (mSv) each year. Participants will have a maximum of three scans and this is approximately equals to ~2.0 µSV per year. At this dose level, no harmful effects of radiation have been demonstrated, as any effect is too small to measure. This risk is believed to be very low.

The scanner will record information for your whole body. During the scanning, we will need to ensure that you are lying on the table in a comfortable position, and it is very important to keep in a very still position. The scanner may produce some noise, earplugs can be offered to reduce the noise when necessary. Some people may experience symptoms of claustrophobia from lying in a confined space. If you do experience discomfort at any time during the scan, you can notify the staff straightway to stop the scanning.

Metal objects can pose a safety risk, so it is important that metal objects are not being worn during the scan. We will thoroughly examine you to make sure there is no reason for you not to have the scan. You must tell us if you have metal implanted in your body, such as pacemaker or metal pins. A DEXA scan information sheet will be provided to you for further information.

The scans we are taking are for research purposes. They are not intended to be used like scans taken for a full clinical examination. The scans will not be used to help diagnose, treat or manage a particular condition. A specialist will look at your DEXA scan for features relevant to the research project. On rare occasions, the specialist may find an unusual feature that could have a significant risk to your health. If this happens, we will contract you to talk about the findings. We cannot guarantee that we will find any / all unusual features.

**8 What if I withdraw from this research project?**

If you do consent to participate, you may withdraw at any time. If you decide to withdraw from the project, please notify a member of the research team.

If you decide to leave the research project, the researchers will not collect additional personal information from you, although personal information already collected will be retained to ensure that the results of the research project can be measured properly. You should be aware that data collected up to the time you withdraw will form part of the research project results. If you do not want your data to be included, you must tell the researchers when you withdraw from the research project.

**9 What happens when the research project ends?**

When the research project is completed, the results will be analysed and synthesised into summary using plain language. The identity of all participants will remain anonymous, and all personal identifications will be removed from the report of findings. The findings will be published in scientific journals or presented to scientific conferences.

All participants will receive a copy of the summary of the findings via email.

Participants who have been allocated to the electroacupuncture only group will receive a copy of the pre-recorded mindfulness meditation audio for self-practise at the end of the study upon request.

Participants who have received sham electroacupuncture will be informed at the end of the study and will be offered a real electroacupuncture treatment if the result shows that electroacupuncture is effective for weight loss. However, they are not obligated to accept the offer.

**How is the research project being conducted?**

**10 What will happen to information about me?**

By signing the consent form you consent to the research team collecting and using information from you for the research project. Any information obtained in connection with this research project that can identify you will remain confidential. All personal information and records that you have provided during the clinical trial will be retained in your participant file and safely locked in the filing cabinet of investigator office. This information will also be entered in a password protected computer for data analysis. Access to this information will only be the individuals listed in this application, or the authorised members of our research team who may also be inspecting your records in the event of data audition. Upon the completion of the study, all records will be kept in the RMIT University, Chinese medicine clinical trial storage room. The information will be retained for 15 years and then will be shredded and disposed as required by the National Health and Medical Research Council (NHMRC) privacy policy.

Your information will only be used for the purpose of this research project, and it will only be disclosed with your permission, except as required by law.

It is anticipated that the results of this research project will be published and/or presented in a variety of forums. In any publication and/or presentation, your personal information will not be disclosed and your identity will not be revealed.

In accordance with relevant Australian and/or Victorian privacy and other relevant laws, you have the right to request access to the information about you that is collected and stored by the research team. You also have the right to request that any information with which you disagree be corrected. Please inform the research team member named at the end of this document if you would like to access your information.

Any information that you provide can be disclosed only if (1) it is protect you or others from harm, (2) if specifically allowed by law, (3) you provide the researchers with written permission. Any information obtained for the purpose of this research project that can identify you will be treated as confidential and securely stored.

**11 Who is organising and funding the research?**

This research project is being conducted by Dr. George Binh Lenon, Dr. Angela Wei Hong Yang ,Dr. Alexander De Foe and Ms Ching Yee Chung.

RMIT University may benefit financially from this research project if, for example, the project assists RMIT University in any commercial enterprise.

You will not benefit financially from your involvement in this research project even if, for example, knowledge acquired from your information proves to be of commercial value to RMIT University.

In addition, if knowledge acquired through this research leads to discoveries that are of commercial value to RMIT University, the researchers or their institutions, there will be no financial benefit to you or your family from these discoveries.

No member of the research team will receive a personal financial benefit from your involvement in this research project (other than their ordinary wages).

**12 Who has reviewed the research project?**

All research in Australia involving humans is reviewed by an independent group of people called a Human Research Ethics Committee (HREC). This research project has been approved by the RMIT University HREC.

This project will be carried out according to the *National Statement on Ethical Conduct in Human Research* (2007). This statement has been developed to protect the interests of people who agree to participate in human research studies.

**13 Further information and who to contact**

The person you may need to contact will depend on the nature of your query. If you want any further information concerning this project or if you have any problems which may be related to your involvement in the project, you can contact the researcher on 03 9925 7584 or any of the following people. The contact details of our team counsellor are also listed below, for any psychological issue related to our treatment provided.

**Research contact person**

| Name | Ms. Ching Yee Chung |
| --- | --- |
| Position | Research student |
| Telephone | 61 0490 454 044 |
| Email | s2006789@student.rmit.edu.au |

**Research contact person**

| Name | Dr. George Binh Lenon |
| --- | --- |
| Position | Chief investigator / Senior supervisor |
| Telephone | 61 3 9925 7175 |
| Email | George.lenon@rmit.edu.au |

**Research contact person**

| Name | Dr. Angela Wei Hong Yang |
| --- | --- |
| Position | Associate investigator / Associate supervisor |
| Telephone | 61 3 9925 6587 |
| Email | Angela.yang@rmit.edu.au |

**Research contact person (Team Counsellor)**

| Name | Dr. Alexander De Foe |
| --- | --- |
| Position | Associate investigator / Associate supervisor |
| Telephone | 61 3 9925 3294 |
| Email | alexander.defoe@rmit.edu.au |

**14 Complaints**

If you have any complaints about any aspect of the project, the way it is being conducted or any questions about being a research participant in general, then you may contact:

| Reviewing HREC name | RMIT University |
| --- | --- |
| HREC Secretary | Peter Burke |
| Telephone | 03 9925 2251 |
| Email | [human.ethics@rmit.edu.au](mailto:human.ethics@rmit.edu.au) |
| Mailing address | Research Ethics Co-ordinator  Research Integrity Governance and Systems  RMIT University  GPO Box 2476  MELBOURNE VIC 3001 |

**Consent Form**

| **Title** | The clinical evaluation of electroacupuncture combined with mindfulness meditation in the weight management |
| --- | --- |
| **Protocol Number** | TBA |
| **Chief Investigator/Senior Supervisor** | Dr. George Binh Lenon |
| **Associate Investigator(s)/Associate Supervisors** | Dr. Anggela Wei Hong Yang  Dr. Alexander De Foe |
| **Research Student(s)** | Ms Ching Yee Chung |
|  |  |

**Acknowledgement by Participant**

I have read and understood the Participant Information Sheet.

I understand the purposes, procedures and risks of the research described in the project.

I have had an opportunity to ask questions and I am satisfied with the answers I have received.

I freely agree to participate in this research project as described and understand that I am free to withdraw at any time during the project without affecting my relationship with RMIT University.

I understand that I will be given a signed copy of this document to keep.

|  | | | | | | | |
| --- | --- | --- | --- | --- | --- | --- | --- |
|  | Name of Participant (please print) | |  | |  |  |  |
|  | | | | | | | |
|  | Signature |  | | Date | |  |  |
|  | | | | | | | |

**Declaration by Researcher^†^**

I have given a verbal explanation of the research project, its procedures and risks and I believe that the participant has understood that explanation.

|  | | | | | | |
| --- | --- | --- | --- | --- | --- | --- |
|  | Name of Researcher^†^ (please print) | |  | | |  |
|  | | | | | |  |
|  | Signature |  | | Date |  |  |
|  | | | | | | |

^†^ An appropriately qualified member of the research team must provide the explanation of, and information concerning, the research project.

Note: All parties signing the consent section must date their own signature.
